# Effect of Calcium Stearate in the Mechanical and Physical Properties of Concrete with PCC and Fly Ash as Binders

**DOI:** 10.3390/ma13061394

**Published:** 2020-03-19

**Authors:** Agus Maryoto, Buntara Sthenly Gan, Nor Intang Setyo Hermanto, Rachmad Setijadi

**Affiliations:** 1 Department of Civil Engineering, Universitas Jenderal Soedirman, Jl. Mayjend Sungkono KM 5, Blater, Purbalingga, Central Java 53371, Indonesia; intang_sh@yahoo.com; 2 Department of Architecture, College of Engineering, Nihon University, 1-Nakagawara, Koriyama, Fukushima 9638642, Japan; buntara@arch.ce.nihon-u.ac.jp; 3 Department of Geology Engineering, Jenderal Soedirman University, Jl. Mayjend Sungkono KM 5, Blater, Purbalingga, Central Java 53371, Indonesia; rsetijadi_ianov@yahoo.co.id

**Keywords:** calcium stearate, Portland composite cement, fly ash, water absorption, infiltration of chloride ion, accelerated corrosion

## Abstract

This work aims to study the effect of Ca(C_18_H_35_O_2_)_2_ (calcium stearate) on the properties of concrete by using Portland composite cement (PCC) and fly ash as binders. The calcium stearate content used in the concrete here consists of 0, 1, 5, and 10 kg per m^3^ of concrete volume, or alternatively, 0 to 2.85% by the weight of cement. We have performed several tests for each of the contents, namely, compressive strength, water absorption, chloride ion infiltration, and accelerated corrosion tests. According to the testing, we have found that with the addition of calcium stearate at 1 kg/m^3^ in self-compacting concrete (SCC) with 10% fly ash, the mechanical and physical properties of SCC can be improved significantly when compared to the SCC without fly ash and calcium stearate, resulting in a stable compressive strength, lower water absorption, lower chloride ion infiltration, and lower degree of corrosion attack.

## 1. Introduction

Portland composite cement (PCC) is a hydraulic binder that is milled together with clinker, gypsum, and one or more inorganic materials, or is the result of mixing Portland cement powder and other inorganic powders. These inorganic materials include blast furnace slag, pozzolan, silicate compounds, and limestone, with a total content of inorganic material of 6–35% of the mass of Portland composite cement [1]. PCC is slightly different from ordinary Portland cement (OPC). In the production process, OPC is made without containing other organic ingredients.

Fly ash is the ash that results from burning coal which is used as fuel in steam power plants. This material can be used as a partial replacement for cement in concrete. Fly ash has a particle size that is smaller than the particle size of cement. Because of this physical condition, the use of fly ash in concrete contributes many advantages in improving the physical and mechanical properties of concrete. The physical and mechanical properties of concrete that can be improved include a lower permeability [2], lower water absorption [3,4], increased tensile strength [5], lower abrasion resistance [6], increased durability [7], lower hydration heat during cement hydration, a decrease in cracks because of smaller shrinkage, and a higher compressive strength of the concrete at 28 days [8]. The high SiO_2_ content in fly ash greatly contributes to the increase in the compressive strength of concrete. Silica oxide (SiO_2_) in fly ash reacts with calcium hydroxide (Ca(OH)_2_) to form the additional calcium silicate hydrate mineral referred to as C-S-H (tobermorite) [9]. Calcium hydroxide itself is one of the compounds formed when tricalcium silicate (C3S), dicalcium silicate (C2S), or tricalcium aluminate (C3A) reacts with water (H_2_O). The use of fly ash in concrete as a substitute for cement further increases the amount of C-S-H/calcium silicate hydrate (tobermorite) formed during the cement hydration process. It can reduce cement the consumption by 3.2–5 kg·m^−3^·MPa^−1^ [10]. In the process of cement production, millions of tons of CO_2_ gas (a pollutant) is released because of combustion to make clinker. Reducing cement consumption in concrete consequently decreases the CO_2_ emissions. Besides that, the appearance of fly ash in concrete also raises the alkalinity of concrete [11]. The higher the alkalinity of concrete, the greater the passive layer protected steel bar in concrete is from corrosion attack.

Not only additives such as fly ash, slag, and silica fumes, but superplasticizers such as water reducers [12] in concrete also can reduce the capillaries and pores [13] in concrete. Although the water/cement ratio used in concrete is very small, the workability of fresh concrete remains good, and this is easily maintained by adding a superplasticizer to the concrete [14]. Because of the low water/cement ratio used, autogenous shrinkage is reduced. Other effects include increasing the elastic modulus [15] and raising the anti-carbonation of the concrete [16].

A large contact angle makes it more difficult for water to seep into concrete [17]. As a result, the water absorption of concrete also drops significantly. Furthermore, in general, chloride ions penetrate into concrete along with water because of the greater contact angle. Automatically, concrete-containing large silica particles are more resistant to corrosion attack. On the contrary, in concrete made with the addition of a water-entraining agent [18], chloride ions enter into concrete more easily, especially in young concrete [19,20]. Corrosion attack on the concrete reinforcement surface causes cracks on the surface of concrete and a decrease in bonding force of reinforcement. As an effect, cracks increase the degree of chloride ingress for concrete [21,22] and bond slipping [23] causes the tensile strength of concrete to decrease. Decreased flexural capacities due to corrosion attacks can occur in both reinforced concrete and pre-stressed concrete [24]. A corroded reinforced concrete structure can be repaired using wire ropes mounted on the surface of concrete structures [25].

Millions of reinforced concrete structures are located in corrosive environments. This is a problem throughout the world. To avoid corrosion attack on steel bars in concrete, some studies must be carried out to increase the resistance properties of concrete. Studies on the use of Ca(C_18_H_35_O_2_)_2_ (calcium stearate) in self-compacting concrete (SCC) with PCC as a binder have been carried out by Maryoto et al. in 2018 [26]. It was found that calcium stearate usage can reduce water absorption [27], the infiltration of chloride ions [28], and corrosion attack [26]. Reducing the diameter of pores also decreases chloride ion ingress [29,30]. It has been shown that corrosion attacks occur massively for concrete without calcium stearate, whereas concrete that uses calcium stearate experiences less corrosion. Maryoto et al. [26] conducted a study on the effect of calcium stearate in concrete with PCC as a binder. The results show that the additional of calcium stearate at 1 kg per m^3^ of concrete reduces the corrosion attack of steel bars by around 8%. Although the effect of calcium stearate on concrete with a PCC binder is well-known, the effect of calcium stearate in concrete using PCC and fly ash as a binder has not yet been studied. Therefore, this study aims in determining the effect of calcium stearate on concrete with a PCC binder, combined with fly ash and a superplasticizer.

## 2. Materials and Methods 

The materials used include PCC, crushed stone, sand, water, a superplasticizer, fly ash, calcium stearate, chemical materials (*PT. Aneka Kurnia*, Semarang, Indonesia) to analyze the chloride ion content, the coating material, sodium chloride, sandpaper, cables, steel bars of a 12-mm diameter, an ammonium citrate solution, copper plates, duct tape, a Whatman filter (*PT. Aneka Kurnia*, Semarang, Indonesia), bromic-phenol blue indicator (*PT. Aneka Kurnia*, Semarang, Indonesia), HNO_3_, and Hg(NO_3_)_2_. The crushed stone utilized in this study had a maximum size of 20 mm and was produced from granite cobblestone. The physical properties of calcium stearate are presented in Table 1. The chemical and physical content of PCC and fly ash are shown in Table 2. The physical characteristics of the PCC used are presented in Table 3. The physical properties of the sand and crushed stone are shown in Table 4.

The equipment used for the experiment consisted of concrete mixers, digital scales, a sieve analyzer, glass beakers, a 150-mm in diameter and 300-mm tall cylinder molding (from fibre glass), a cylinder molding (polivinyl chloride pipe), that was 75-mm in diameter and 150-mm tall a cube molding (cast iron) with 150-mm sides, a beam molding of a 100 mm × 100 mm × 200 mm (multiplex wood) size, a direct current power supply (China), a Los Angeles machine (PT. MBT Utama, Jakarta, Indonesia), and a universal testing machine (Physical Test Solution, Culver city, CA, USA).

Based on the physical properties of the cement, fly ash, sand, and crushed stone, the concrete mixtures were designed as shown in Table 5. Percentage of materials in the Tabel 5 are ratio between those material and weight of cement. 

The superplasticizer content used was 0.25% by weight of cement, and the amount of fly ash used was 10% by weight of cement. In Table 5, the symbols G2, G3, and G4 indicate that the quality of the concrete used was 20, 30, and 40 MPa, respectively. The symbols S0 and S1 show that the superplasticizer used was 0% and 0.25% by weight of cement. The symbols F0 and F1 show that the fly ash content was 0% or 10% by weight of the cement. The symbols C0, C1, C5, and C10 show that the calcium stearate used in the mix proportion is 0, 1, 5, and 10 kg per m^3^ of concrete, respectively. When converted into percentages by the weight of cement, the values are 0.28, 1.43, 2.85, 0.24, 1.20, 2.40, 0.19, 0.93, and 1.86%.

### 2.1. Specimens and Type of Testing

The tests conducted in the laboratory include compressive strength, water absorption, infiltration of chloride ions, and accelerated corrosion tests. The number of specimens for each code mix proportion was three pieces. Specifically, for the infiltration of chloride ion specimens, each specimen was investigated for the chloride ion content at depths of 1, 2, 4, 6, and 8 cm from the surface of the test specimen, and each test was taken with three samples. The compressive strength specimen was cylindrical, with a diameter of 150 mm and a height of 300 mm. The water absorption specimens were cylinders with a diameter of 75 mm and a height of 150 mm. The infiltration of chloride ions and the accelerated corrosion test specimens were cubes side lengths of 150 mm and beams with a size of 100 × 100 × 200 mm, respectively. The number of specimens for each code for the compressive strength, water absorption, and accelerated corrosion tests was 3 pieces. Meanwhile, the number of specimens for the infiltration of chloride ions test was 15 pieces.

### 2.2. Specimens Production

**Figure 1:**
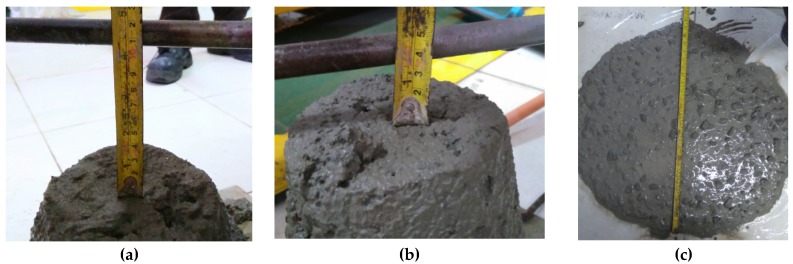
Workability of concrete: (**a**) Mix proportion of concrete without superplasticizer; (**b**) mix proportion of concrete with superplasticizer before addition; (**c**) after adding the superplasticizer.

**Figure 2:**
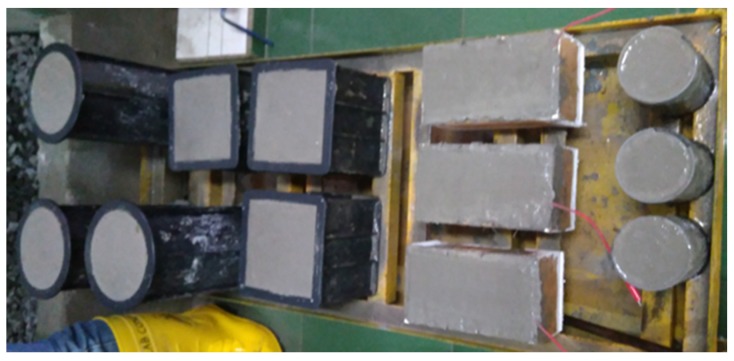
Molds of the specimens after being filled with fresh concrete.

The crushed stone and sand used in the production of the fresh concrete were kept in saturated surface dry conditions. They were weighed according to the mix design and were put in a concrete mixer and then mixed. The next step was that cement and fly ash (for concrete using fly ash) were inserted and mixed again with a concrete mixer. The next step was to add water to the concrete mixer and mix it again, such that the fresh concrete became homogeneous. For concrete using a superplasticizer, after the fresh concrete was quite homogeneous, the superplasticizer was put into the fresh concrete and then mixed again. Workability testing was then carried out with a slump test before the fresh concrete was poured into a mold. The average workability of concrete without the superplasticizer was around 10 cm. Further, the concrete with the superplasticizer had a workability of 3 cm before the superplasticizer added, and a 65-cm diameter for workability after the superplasticizer was added (Figure 1a–c). Especially for the accelerated corrosion test, the fresh concrete is poured into the concrete after the steel bar and cables to generate artificial corrosion in the mold. Figure 2 shows the moldings after being filled with fresh concrete.

### 2.3. Testing Protocol

**Figure 3:**
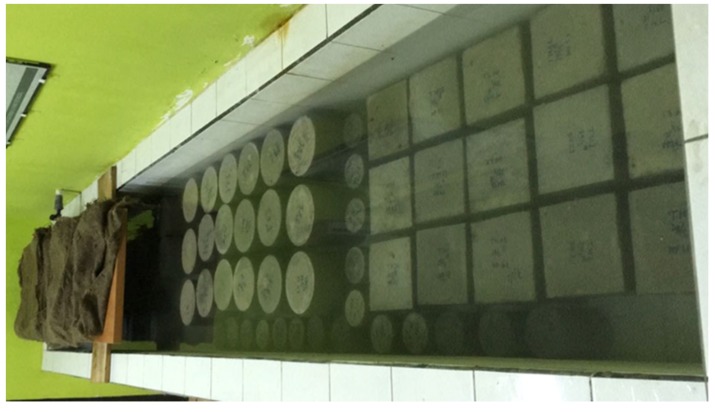
Process of curing for specimens.

After the concrete was one day old, the molds were removed, and all specimens were treated by immersing them in water, except for the accelerated corrosion specimen (Figure 3). The accelerated corrosion specimens were treated by covering them with a wet mattress (see Figure 3 on the left side). Curing of the concrete specimens was carried out for 27 days. The temperature of the water ponds was around 27.9 °C and the humidity was 76%. The test objects stopped water curing at 28 days.

#### 2.3.1. Compressive Strength

The compressive strength test was conducted based on the ASTM C39-94 standard [31]. The compressive strength specimens were placed in an open space and protected from sunlight until the concrete surface became dry. To ensure that the working load was spread evenly, the specimen surfaces were coated with a sulfur capping. The compressive strength test subjects were put in the middle of a universal testing machine (UTM). The UTM was turned on, with speeds ranging from 2 to 4 kg/cm^2^ per second. The maximum force that was applied when the test object ruptured was noted. The compressive strength can be calculated by dividing the maximum force by the area of the test object.

#### 2.3.2. Water Absorption

**Figure 4:**
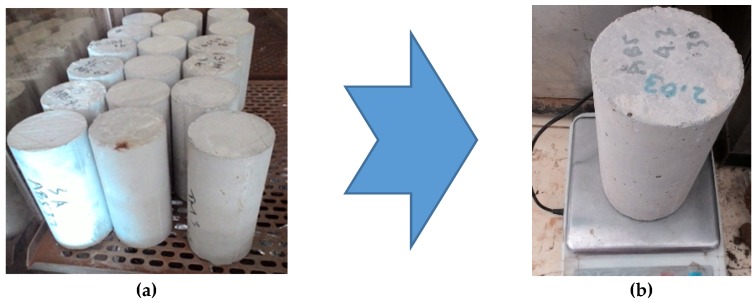

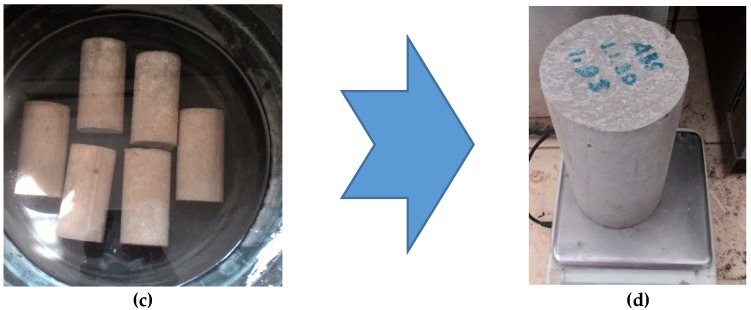
Processes of water absorption testing: (**a**) Drying the specimens; (**b**) weighing a dry specimen; (**c**) soaking the specimens in water; (**d**) weighing a saturated surface dry condition specimen.

After the water absorption specimens were 28 days old, the specimens were dried in an oven for 3 days at a temperature of 100 ± 5 °C [32]. The specimens were weighed in dry conditions such that the dry weight was obtained. The next step was to soak the specimens in water for 10 min. The specimens were weighed under saturated surface dry conditions and the wet weight was obtained. Water absorption can be calculated using Equation (1). The water absorption testing process can be seen in Figure 4.

(1) $$\mathrm{Water}\;\mathrm{absorption}\;(\%)=\frac{W_{wet}-W_{dry}}{W_{dry}}\times 100\%$$

#### 2.3.3. Infiltration of Chloride Ions

**Figure 5:**
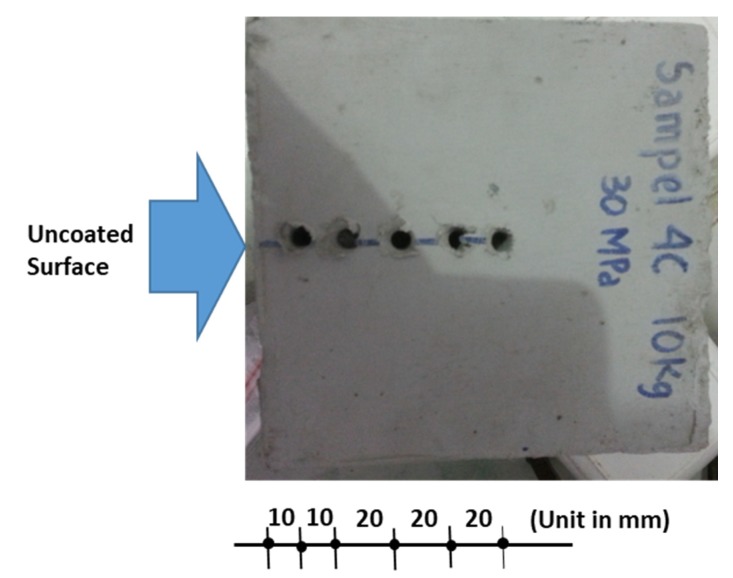
Drilling a specimen for the infiltration of chloride ions test.

After the test subjects were treated in water for 27 days, the 150 × 150 × 150 mm cube specimens were removed from the water pond. When the specimens had dried, five surfaces of each specimen were coated with a waterproof coating. Only one surface was left without a waterproof surface coating. The next step was to soak the specimens in sea water containing 3% sodium chloride with water depths as high as 3 cm from the top concrete surface for 90 days after the waterproof coating was dry. The specimens were removed from the sea water pond when they were 90 days old and were then left to dry in an open space. The specimens were then drilled at distances of 1, 2, 4, 6, and 8 cm from the surface of the unprotected surface in a dry condition, as in Figure 5.

**Figure 6:**
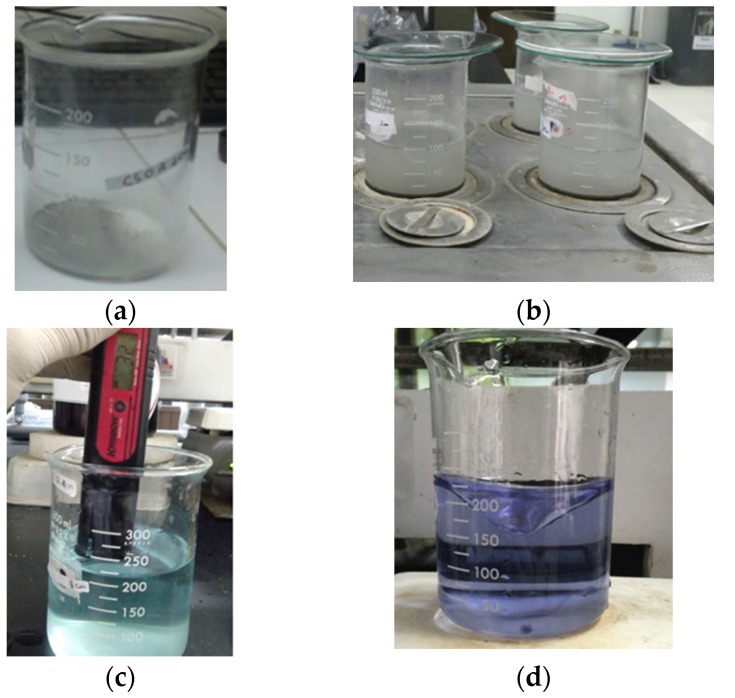
Processes of chloride ion infiltration testing in the concrete: (**a**) Concrete powder in glass beaker; (**b**) heating the specimen with the steam bath; (**c**) solution pH of 3.2; (**d**) purple solution.

The powder was then analyzed for its chloride ion content using the wet chemical analysis method, which is one of the methods used to analyze the chemical elements in materials using a chemical reaction, usually the decomposition of the sample using a reagent. On the other hand, to analyze the chemical elements, one may also utilize X-ray diffraction (XRD) or dry chemical analysis. Because no liquids are used in the XRD process, it is known as a dry chemical analysis. The procedure for testing the chloride ion content is as follows. Total of 5 grams of powder was weighed and put into a glass beaker (Figure 6a). Then 100 mL of water was added and heated in a steam bath for 1 hour (Figure 6b) and then mixed well. It was then filtered with a Whatman filter paper number 41 and then the product was transferred to a glass beaker. The sediment was cleaned from the Whatman filter paper with water and transferred into a glass beaker until 200 cubic centimeter volume is reached. Then 4 drops of a bromic-phenol blue indicator was added into the solution until the solution changed color to red (like the color of a clay brick). Then, HNO_3_ was added until the color of the solution was green and the pH was around 3.2 (see Figure 6c). The solution was then titrated using Hg(NO_3_)_2_ at 0.14 N until the color changed to purple (Figure 6d). The chloride ion content was then calculated by Equation (2).

(2) $${\mathrm{Cl}}^{¯}=\frac{f\times V}{5000}\times 100\%,$$

where *f* is the equality between H(NO_3_)_2_ and Cl^−^ (mg Hg(NO_3_)_2_/ cc Cl^−^) and *V* is the volume of a titrant (volume (in drops) of Hg(NO_3_)_2_ subtracted by blank volume (in drops) of Hg(NO_3_)_2_.

#### 2.3.4. Accelerated Corrosion

**Figure 7:**
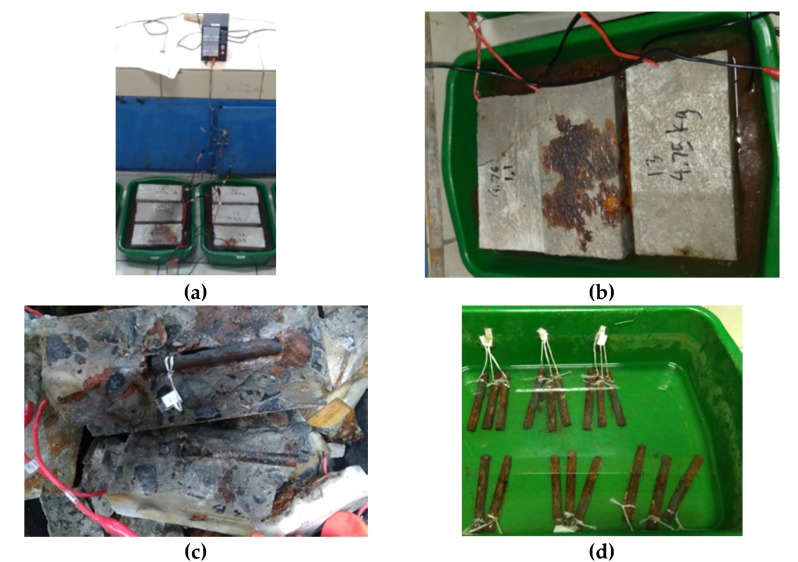
Processes of accelerated corrosion: (**a**) Accelerated corrosion; (**b**) NaCl solution changed to be brown in color; (**c**) removal of the corroded steel bar; (**d**) soaking the corroded steel bar in ammonium citrate.

The accelerated corrosion test was carried out when the concrete was 28 days old. The process for the accelerated corrosion testing is listed as follows. The specimens were immersed in water containing 3% sodium chloride. The water level was 90 mm from the bottom surface of the specimen or 10 mm lower than the top concrete surface. For example, six pieces of 20 MPa quality concrete specimens with the G2S0F0C0, G2S1F0C0, G2S1F1C0, G2S1F1C1, G2S1F1C5, and G2S1F1C10 codes were connected in parallel with one direct power supply (see Figure 7a). This was done so that the voltage used to generate corrosion in the specimens with different codes was the same. The voltage used in this study was 22.5 V. A positive current from a direct power supply was connected to the steel bar in the concrete, and a negative current was connected to a copper plate placed in the solution with 3% NaCl, which was located under the specimens. Then, the direct power supply was turned on to generate accelerated corrosion. This test was carried out for 21 days. The corrosion that arose could be investigated through the cracked concrete surfaces, and the solution turned brown (see Figure 7b). The power supply was turned off when 21 days had elapsed since the power supply was turned on. The test objects were broken down to remove the corroded reinforcement (Figure 7c). The corroded reinforcement was cleaned with a wire brush and then the steel bar was immersed in a 10% ammonium citrate solution for 24 hours. After the reinforcement was clean from the corrosion product, we weighed the reinforcement (*W*_2_) (Figure 7d). The amount of corrosion that occurred was calculated by subtracting the weight of *W*_2_ from the initial reinforcement weight (*W*_1_) before corrosion. The corrosion level calculation was carried out using Equation (3).

(3) $$\mathrm{Corrosion}\;(\%)=\frac{W_{1}-W_{2}}{W_{1}}\times 100\%$$

## 3. Results

It is important to note here that we use a red box in the figures below to identify a concrete test result where calcium stearate has been involved. The first two symbols of G2, G3, and G3 are omitted in the figures and have been replaced by the symbol “--“, and the legends show explanations of 20, 30, and 40 MPa with different line colors. 

### 3.1. Compressive Strength

**Figure 8:**
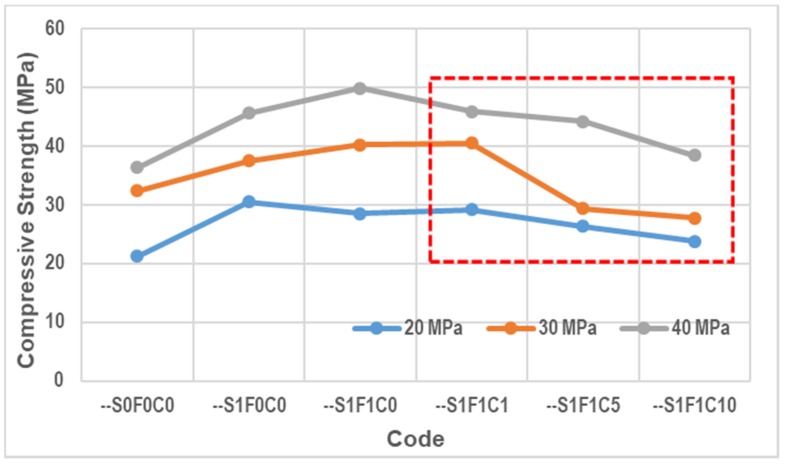
Compressive strength test results.

The results of compressive strength testing are shown in Figure 8. We used an average of three test pieces here. The vertical axis shows the compressive strength in MPa units and the horizontal axis represents the codes of the specimens. 

### 3.2. Water Absorption

**Figure 9:**
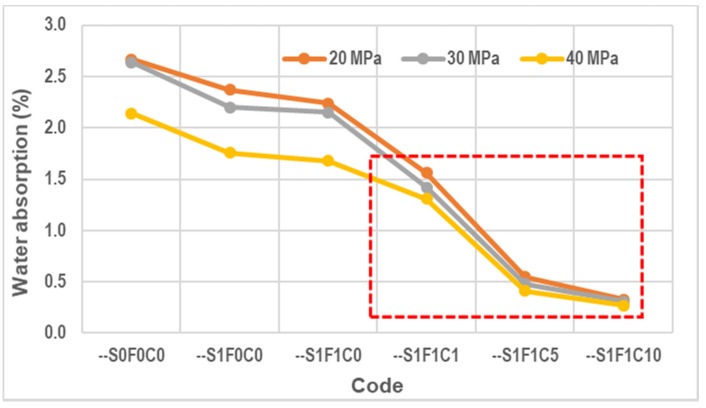
Water absorption of concrete.

The water absorption results are shown in Figure 9. The horizontal axis shows representations of the different concrete mixture types. The water absorption is represented by the vertical axis.

### 3.3. Infiltration of Chloride Ion

**Figure 10:**
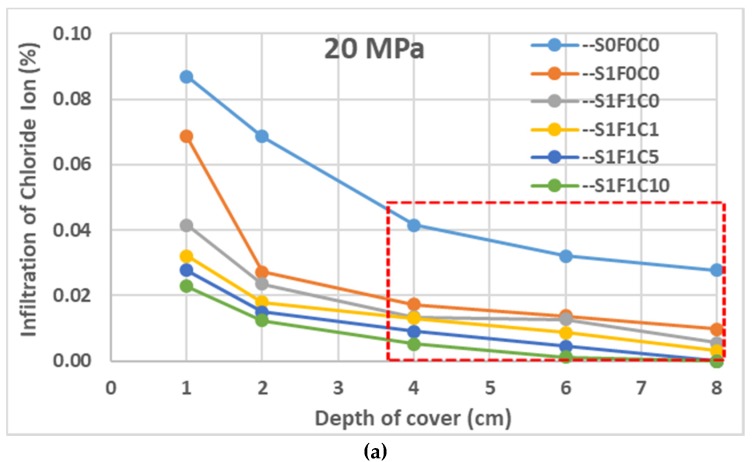

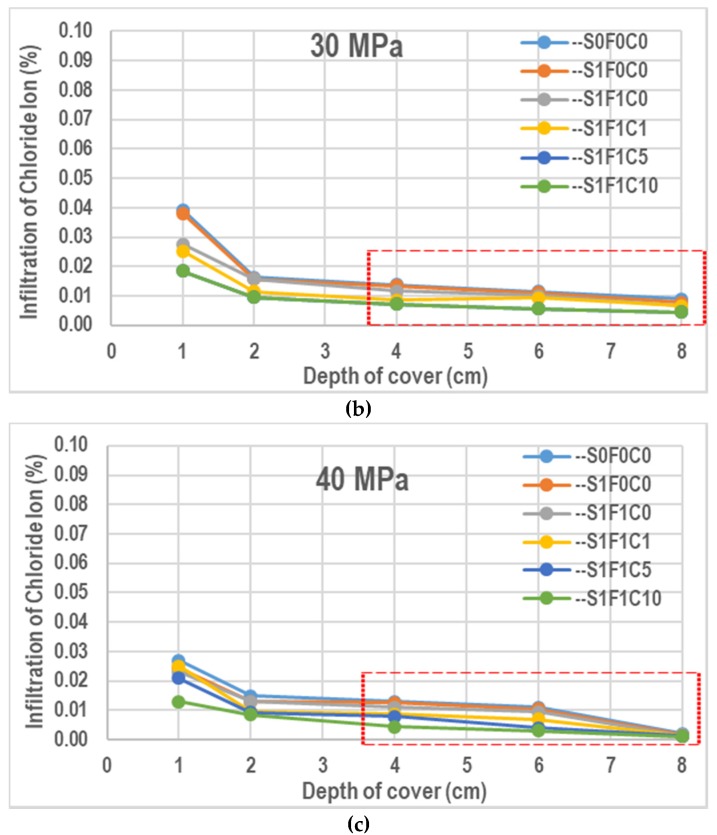
Infiltration of chloride ions in the concrete: (**a**) 20 MPa; (**b**) 30 MPa; (**c)** 40 MPa.

Figure 10 shows the relationship between the thickness of the concrete cover and the amount of chloride ion infiltration. Figure 10a–c shows the amount of chloride ion infiltration in concrete of 20, 30, and 40 MPa quality.

### 3.4. Accelerated Corrosion

**Figure 11:**
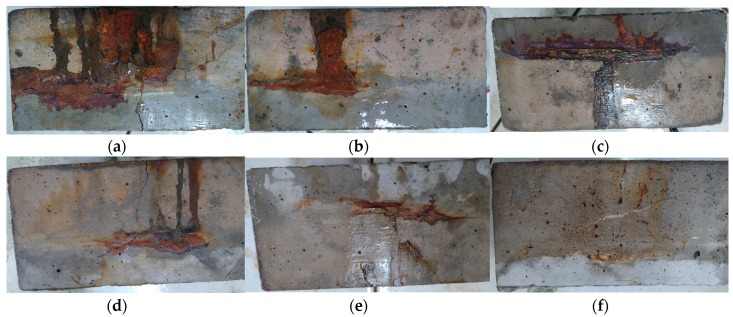
Appearance of corrosive products on 20 MPa concrete specimens: (**a**) G2S0F0C0; (**b**) G2S1F0C0; (**c**) G2S1F1C0; (**d**) G2S1F1C1; (**e**) G2S1F1C5; (**f**) G2S1F1C10.

Figure 11 shows a 20 MPa concrete specimen with accelerated corrosion for 21 days. During the process of accelerated corrosion, the NaCl solution changed from a clear color to be a reddish orange color. Then, this was followed by cracks on the surfaces of the specimens. Finally, the corrosion product was pressed out from the surface of the reinforcement to the surface of the concrete.

**Figure 12:**
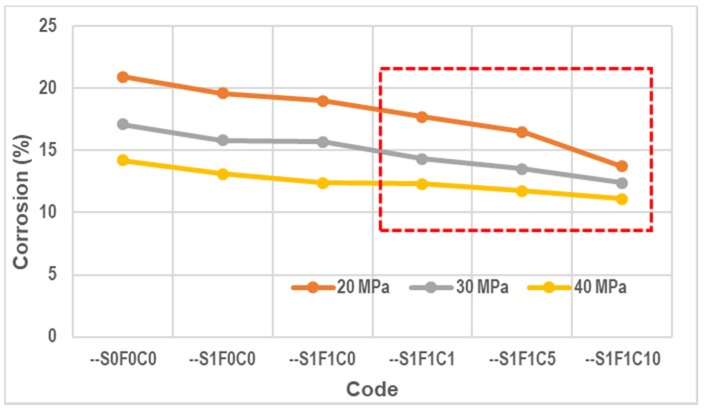
Corrosion attack in various concrete mix proportions.

The accelerated corrosion specimens were then broken down and the corroded reinforcement was removed. Both the amount of corrosion that occurred in the specimens for each code and the quality of the concrete are shown in Figure 12. The results in Figure 12 show the average corrosion results of three specimens.

## 4. Discussion

### 4.1. Compressive Strength

The compressive strength of specimens --S1F1C1, --S1F1C5, and --S1F1C10 began to decrease when compared to specimen number --S1F1C0. This is due to the addition of calcium stearate at 1, 5, and 10 kg in the concrete. Calcium stearate reacts with cement and water to form a wax-like constituent. This compound has a weaker bond than the bond formed by the C-S-H compound. Therefore, the wax-like constituents in the concrete cause the compressive strength to decrease [30]. This result matches that of the study carried out by Chari et al. [27]. By photomicrograph analyses [33], it has been shown that calcium stearate and cement have weaker bonding. However, the reduced compressive strength of concrete due to the use of calcium stearate is an effect that is not too significant when the calcium stearate used is only used at 1 kg per m^3^ of fresh concrete. So, this content may still safely be used in concrete with a binder in the form of SCC added with fly ash and a superplasticizer as well.

By comparing SCC (self-compacting concrete has high workability, usually designed by adding a superplasticizer) without fly ash, in this case the code is --S1F0C0, the compressive strength of the SCC with calcium stearate and fly ash (in this case the codes are --S1F1C1, --S1F1C5, and --S1F1C10) is not significantly different. This shows that the presence of calcium stearate in SCC with fly ash has a good impact in the compressive strength of SCC.

### 4.2. Water Absorption

**Figure 13:**
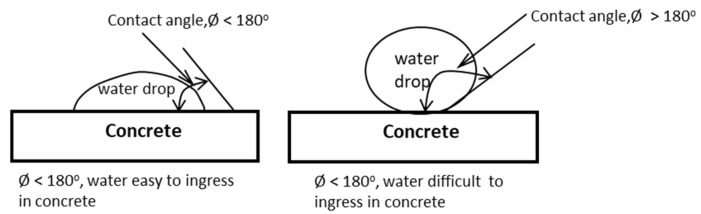
Contact angle of water and the concrete surface.

Specimens --S1F1C1, --S1F1C5, and --S1F1C10 are concrete specimens using calcium stearate at 1, 5, and 10 kg per m^3^ as an additive material. It can be observed that water absorption decreases significantly compared to concrete without calcium stearate (codes --S0F0C0, --S1F0C0, and --S1F1C0). This decrease occurred in all qualities of concrete here when using calcium stearate. This could be explained by the possibility that calcium stearate reacts with cement and water to form a wax-like compound. This wax-like compound coats the surface of capillaries during the evaporation process. This compound is hydrophobic. As a result of this hydrophobic nature, the contact angle that forms between water and cement becomes large. The contact angle of water and concrete is usually lower than 180°. SCC with calcium stearate has a contact angle of more than 180°, as shown in Figure 13 [34]. Thus, it is difficult for water to get into the concrete. This result shares the same tendency as in the work of Chari et al. [27]. It shows that the addition of calcium stearate at 1 kg/m^3^ of concrete decreases water absorption by around 20% after 30 minutes of immersion in water.

### 4.3. Infiltration of Chloride Ion

Figure 10 shows the tendency of chloride ion infiltration for each concrete cover depth. Figure 10a–c shows the relationship between the amount of chloride ion infiltration and the mix proportion of concrete used. The addition of a superplasticizer, fly ash, and calcium stearate also has a good influence on the properties of concrete in the degree of chloride ion infiltration. The greater the calcium stearate added to the concrete, the lower the chloride ion infiltration. The infiltration of chloride ions has a similar tendency to water absorption for concrete. The infiltration of chloride ions is reduced because of the addition of a superplasticizer in concrete (code --S1F0C0). This value is decreased again when fly ash is added in the concrete along with a superplasticizer (code --S1F1C0). Finally, the level of chloride ion infiltration in concrete reduces significantly due to the addition of calcium stearate at 1, 5, and 10 kg per m^3^ of concrete (codes --S1F1C1, --S1F1C5, and --S1F1C10). Calcium stearate has a high degree of swelling when it contacts water and forms a gel. The gel functions as a power blocker and blocks capillaries when other liquids, together with chloride ions, penetrate into the concrete. Another influence is that the addition of calcium stearate makes the surfaces of capillaries have a hydrophobic effect [28].

### 4.4. Accelerated Corrosion

The concrete without a superplasticizer, fly ash, and calcium stearate (code --S0F0C0) had the highest degree of corrosion attack, contrary to the specimen of concrete with a superplasticizer as a water reducer, fly ash, and calcium stearate (codes --S1F1C1, --S1F1C5, and --S1F1C10). The addition of calcium stearate clearly can significantly reduce corrosion attack in concrete with a PCC binder, fly ash, and a superplasticizer. This result is in accordance with the work of Quraishi et al. from 2011 [33]. It is because calcium stearate prevents corrosion due to adsorption on the steel surface through polar carboxylate group and by blocking the pores, forming an insoluble hydrophobic surface on the steel. As the result, it reduces the infiltration of chloride ions, carbon dioxide, moisture, and other aggressive agent into the concrete. The tendency of decreasing corrosion attack on the concrete tested here has the same trend as decreasing water absorption and the infiltration of chloride ions.

**Figure 14:**
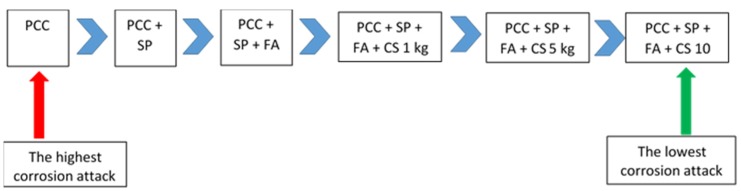
Effect of corrosion attack due to material used in the concrete. PCC: Portland composite cement; SP: Superplasticizer; FA: Fly ash; CS: Calcium stearate. Here, 1 kg, 5 kg, and 10 kg were the dosages of calcium stearate in the concrete.

The diagram in Figure 14 shows the proportion of concrete mixes without sand, crushed stone, and water, from the highest corrosion attack to the lowest.

## 5. Conclusions

When compared with concrete with PCC as a binder at 20, 30, and 40 MPa qualities, the following conclusions can be made based on the fresh concrete without calcium stearate. First, the compressive strength of the concrete when adding calcium stearate is relatively stable. The compressive strength of concrete containing calcium stearate is not significantly different when compared to concrete without a superplasticizer, fly ash, and calcium stearate. Moreover, the use of calcium stearate will clearly reduce the water absorption capability of concrete and the infiltration of chloride ions. Finally, our tests have revealed that corrosion of concrete can be reduced by using calcium stearate. Hence, calcium stearate is a good candidate to improve the mechanical and physical properties of concrete. Overall, the use of calcium stearate in SCC-containing fly ash has a beneficial effect on its properties; the use of calcium stearate increases compressive strength, reduces water absorption, decreases the infiltration of chloride ions, and diminishes the degree of corrosion attack. In the next study we will discuss the effect of calcium stearate on the bonding force between steel bars and concrete.

## Figures and Tables

**Table 1:** Physical properties of calcium stearate [30].

Physical Characteristic	Analysis
Chemical formula	Ca(C_18_H_35_O_2_)_2_
Appearance	Fine white powder
Melting point	120 °C
Free fatty acid	Maximum of 0.5%
Loss on drying	Maximum of 2%
Metal content	0.65 (ppm)
Specific gravity	0.25

**Table 2:** Chemical content of the Portland composite cement (PCC) and fly ash. LOI: loss of ignition; AE: Alkali equivalence; IR: insoluble residue.

Chemical and Physical Composition	SiO_2_	Al_2_O_3_	Fe_2_O_3_	CaO	MgO	SO_3_	LOI	AE	IR
PCC (%)	19.6	5.84	3.93	64.55	1.26	2.31	1.32	0.63	0.56
Indonesia National Standard for PCC	-	-	-	-	Max. of 6.0	Max. of 4.0	Max. of 5.0	0.6	Max. of 3.0
Fly ash (%)	46.21	18.97	10.13	9.85	2.76	0.18	0.20	-	7.5

**Table 3:** Physical properties of the PCC.

Physical Characteristic	Result of Testing	SNI 15-7064-2004 (PCC)
Fineness with Blaine apparatus (m^2^/kg)	396	Min. of 280
Fineness with a 45-µm mesh of residue (%)	10.70	-
Initial setting time (minutes)	130	Min. of 45
Final setting time (minutes)	263	Max. of 375
Expansion (%)	0.04	Min. of 0.80
Compressive strength at 3 days (MPa)	19.2	Min. of 12.5
Compressive strength at 7 days (MPa)	24.8	Min. of 20.0
Compressive strength at 28 days (MPa)	32.1	Min. of 25.0
False set (minutes)	46.21	Min. of 50

**Table 4:** Physical properties of the sand and crushed stone.

Physical Properties	Sand	Crushed Stone
Specific gravity	2.60	2.57
Water absorption (%)	3.40	2.50
Density (tons/m^3^)	1.57	1.48
Fineness modulus	2.80	6.72
Clay content (%)	6.72	1.35

**Table 5:** Mix proportion of concrete at 20, 30, and 40 MPa.

Materials	Code of Concrete at 20 MPa					
	G2S0F0C0	G2S1F0C0	G2S1F1C0	G2S1F1C1	G2S1F1C5	G2S1F1C10
Cement (kg)	350	350	350	350	350	350
Fly ash (%)	0	0	10	10	10	10
Sand (%)	251.43	251.43	251.43	251.43	251.43	251.43
Crushed stone (%)	244.29	244.29	244.29	244.29	244.29	244.29
Water (%)	58.57	50.00	50.00	50.00	50.00	50.00
Superplasticizer (%)	0.00	0.25	0.25	0.25	0.25	0.25
Calcium stearate (%)	0.00	0.00	0.00	0.29	1.43	2.86
	**Code of Concrete at 30 MPa**					
	**G3S0F0C0**	**G3S1F0C0**	**G3S1F1C0**	**G3S1F1C1**	**G3S1F1C5**	**G3S1F1C10**
Cement (kg)	415	415	415	415	415	415
Fly ash (%)	0	0	10	10	10	10
Sand (%)	189.16	189.16	189.16	189.16	189.16	189.16
Crushed stone (%)	214.46	214.46	214.46	214.46	214.46	214.46
Water (%)	51.08	45.06	45.06	45.06	45.06	45.06
Superplasticizer (%)	0	0.25	0.25	0.25	0.25	0.25
Calcium stearate (%)	0	0	0	0.24	1.20	2.41
	**Code of Concrete at 40 MPa**					
	**G4S0F0C0**	**G4S1F0C0**	**G4S1F1C0**	**G4S1F1C1**	**G4S1F1C5**	**G4S1F1C10**
Cement (kg)	535	535	535	535	535	535
Fly ash (%)	0	0	10	10	10	10
Sand (%)	140.19	140.19	140.19	140.19	140.19	140.19
Crushed stone (%)	162.62	162.62	162.62	162.62	162.62	162.62
Water (%)	39.25	33.64	33.64	33.64	33.64	33.64
Superplasticizer (%)	0	0.25	0.25	0.25	0.25	0.25
Calcium stearate (%)	0	0	0	0.19	0.93	1.87
